# Ubiquitin in Influenza Virus Entry and Innate Immunity

**DOI:** 10.3390/v8100293

**Published:** 2016-10-24

**Authors:** Alina Rudnicka, Yohei Yamauchi

**Affiliations:** 1 Institute of Molecular Life Sciences, University of Zurich, Winterthurerstrasse 190, Zurich CH-8057, Switzerland; alinaewa.rudnicka@uzh.ch; 2 School of Cellular and Molecular Medicine, University of Bristol, Biomedical Sciences Building, University Walk, Bristol BS8 1TD, UK; 3 Structural Biology Research Center, Department of Biological Science, Graduate School of Science, Nagoya University, Furo-cho, Chikusa-ku, Nagoya 464-8602, Japan

**Keywords:** ubiquitin, unanchored ubiquitin, HDAC6, aggresome processing, influenza virus, virus entry, virus uncoating, innate immunity, virus–host interactions

## Abstract

Viruses are obligatory cellular parasites. Their mission is to enter a host cell, to transfer the viral genome, and to replicate progeny whilst diverting cellular immunity. The role of ubiquitin is to regulate fundamental cellular processes such as endocytosis, protein degradation, and immune signaling. Many viruses including influenza A virus (IAV) usurp ubiquitination and ubiquitin-like modifications to establish infection. In this focused review, we discuss how ubiquitin and unanchored ubiquitin regulate IAV host cell entry, and how histone deacetylase 6 (HDAC6), a cytoplasmic deacetylase with ubiquitin-binding activity, mediates IAV capsid uncoating. We also discuss the roles of ubiquitin in innate immunity and its implications in the IAV life cycle.

## 1. Ubiquitin and Ubiquitination

Ubiquitin is a small, 8.5 kDa protein composed of 76 amino acids expressed in different tissues and present in different subcellular compartments. Post-translational attachment of ubiquitin to other proteins, namely ubiquitination, alters the function, location, or trafficking of the protein, or targets it for destruction by the 26S proteasome. The ability of ubiquitin to form structurally and functionally distinct polymers greatly increases the complexity of ubiquitination. Ubiquitin has a globular shape with the last four C-terminal residues (LRGG) extending from the compact structure. C-terminal glycine (G) can be covalently conjugated to proteins by isopeptide linkage to the ε-amino group of lysine (K) residues or less frequently to the N-terminal α-amino group or the thiol group of cysteine residues. Ubiquitin itself contains eight amino groups to which another ubiquitin molecule can be conjugated: the ε-amino groups of seven K residues (K6, 11, 27, 29, 33, 48 and 63) and the α-amino group of the N-terminal methionine residue. All of the eight ubiquitin chain types are present in the cell, among which the K48- and K63-linked chains are most abundant and the best described. K48-based linkages lead mainly to the proteasome-mediated degradation of the ubiquitinated protein, while K63-based chains control primarily protein endocytosis, trafficking, and enzyme activity [1,2,3,4].

**Figure 1:**
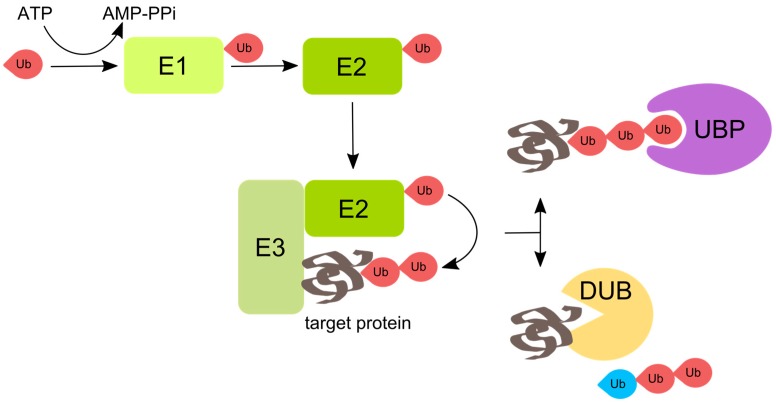
Enzymatic pathway of protein ubiquitination. The attachment of ubiquitin (Ub) to proteins involves consecutive action of three classes of enzymes: ubiquitin-activating enzyme E1, ubiquitin-conjugating enzyme E2, and ubiquitin ligase E3. First, the C-terminus of ubiquitin binds to E1 in an ATP-dependent manner. Ubiquitin is then transferred from the E1 to E2. Finally, the E3 binds both the ubiquitin-bound E2 and the substrate and catalyzes formation of an isopeptide bond between the C-terminus of ubiquitin and the substrate lysine residue. The lysines on the substrate-conjugated ubiquitin can be further polyubiquitinated. A multitude of cellular proteins that contain different ubiquitin-binding domains—namely ubiquitin binding proteins (UBPs)—mediate the cellular functions of ubiquitination. Deubiquitinases (DUBs) that also contain ubiquitin-binding domains can revert or modify ubiquitination. DUBs vary in the specificity towards different types of polyubiquitin linkages, the position of cleavage within polyubiquitin chains, the ability to separate single ubiquitin moieties, etc. Some DUBs produce unanchored polyubiquitin chains that regulate aggresome processing and innate immunity. The **red** ubiquitin depicts ubiquitin that is anchored, and the **blue** ubiquitin depicts unanchored ubiquitin with a free C-terminus.

Mechanistically, the process of protein ubiquitination involves a three-step enzymatic cascade, which starts with the ubiquitin-activating enzyme E1, followed by the ubiquitin-conjugating enzyme E2, and the ubiquitin ligase E3. Ubiquitin is activated in an ATP-dependent manner, when a high-energy thioester bond is formed between the C-terminus of ubiquitin and an internal cysteine residue of the ubiquitin-activating enzyme E1. Activated ubiquitin is then transferred onto the active site cysteine of one of E2-conjugating enzymes. Finally, the formation of an isopeptide bond is catalyzed by E3 ubiquitin ligases, which link ubiquitin moieties to target proteins or elongate a polyubiquitin chain (Figure 1) [5,6,7].

In complex with E2, the E3 ubiquitin ligase forms an isopeptide bond between ubiquitin moieties or between ubiquitin and substrate protein. In most cases the type of linkage is determined by E2 enzymes, except for the linkage between the amino group of the N-terminal methionine residue, determined by the E3 ubiquitin ligase called linear ubiquitin chain assembly complex (LUBAC) [8]. The E3 ubiquitin ligases determine the substrate specificity of ubiquitination, and the diversity of the cellular functions of ubiquitination is reflected in the existence of hundreds of different E3s in mammals, compared with roughly thirty-five E2s and only two E1s in humans. E3 enzymes are currently classified into three main families with different structural and functional characteristics: the homologous to E6AP C-terminus (HECT) domain family of ubiquitin ligases, the cullin-really interesting new gene (RING) family of ubiquitin ligases, and the U-box containing ubiquitin ligases [2,3,9]. E3 ligases can be single- or multi-subunit enzymes; in the second case ubiquitin-binding and substrate binding domains reside on separate polypeptides brought together by adaptor proteins.

Ubiquitinated substrates are subsequently recognized by a large number of proteins that contain different ubiquitin-binding domains; among these are DUBs, a group of about 100 enzymes in mammals that hydrolyze isopeptide linkages between ubiquitin moieties, or between ubiquitin and the substrate. DUBs do more than cancelling the ubiquitin mark: they differ in substrate and linkage type specificity, and in the position of the linkage in the polyubiquitin chain (between ubiquitin moieties or between ubiquitin and the substrate) [10,11]. Hydrolytic activity of DUBs leads to recycling of mono-ubiquitin in the cell, and to generation of free ubiquitin chains, which regulate aggresome formation and innate immune signaling [12,13].

## 2. Viruses Interact with Ubiquitination

Viruses have evolved a large arsenal of strategies to exploit processes regulated by ubiquitin (for a detailed review, refer to [14,15]). They may target unwanted cellular proteins for degradation by K48-linked polyubiquitination or revert undesirable ubiquitination events through deubiquitination. Some viruses encode their own E3 ubiquitin ligases and DUBs, such as infected cell polypeptide 0 (ICP0) of herpes simplex virus type 1 (HSV-1), a multifunctional protein with RING domain E3 ubiquitin ligase activity [16]. Others subvert the specificity of cellular E3 ubiquitin ligases and DUBs in order to avoid self-degradation.

The importance of ubiquitination for virus infection has also been pointed out in multiple studies based on the treatment of infected cells with proteasome inhibitors. Such a treatment not only blocks the ubiquitin proteasome system (UPS), but also depletes the cellular pool of free ubiquitin, affecting many of the cellular pathways involving ubiquitin. Functional UPS is important for the replication of major human pathogens such as herpesviruses, poxviruses, hepadnaviruses, adenoviruses, influenzaviruses, retroviruses, coronaviruses, paramyxoviruses, picornaviruses and rotaviruses [14]. UPS components are upregulated in primary human airway epithelial cells infected by influenza A virus (IAV) [17]. Proteasome inhibition leads to sequestration of incoming IAV in the cytoplasmic compartments and blocks productive viral entry [18,19].

Ubiquitination also plays an important role in innate immunity to IAV. The host antiviral factor cyclophilin A targets the influenza matrix protein (M1) for ubiquitin-mediated degradation [20], while zinc finger antiviral protein ZAPL promotes ubiquitination and degradation of viral polymerase subunits PA and PB2 [21]. Interferon (IFN)-induced transmembrane protein 3 (IFITM3), a potent antiviral protein which inhibits IAV cytosolic entry [22,23], is downregulated by ubiquitination by E3 ubiquitin ligase neural precursor cell expressed developmentally downregulated protein 4 (NEDD4), the depletion of which boosts cell resistance to IAV infection [24]. Tripartite motif-containing (TRIM) proteins, which belong to the RING family of E3 ubiquitin ligases, counteract IAV replication. For example, TRIM25 is a key mediator in antiviral retinoic acid-inducible gene 1 (RIG-I) signaling [25] (described in Section 7). TRIM22 and TRIM32 ubiquitinate nucleoprotein (NP) and viral polymerase subunit PB1, respectively [26,27], targeting them for degradation. IAV proteins indirectly modulate ubiquitination events to evade immune responses and support virus replication. For example, nonstructural protein 1 (NS1) inhibits TRIM25 [28]. Expression of viral hemagglutinin (HA) in cells causes phosphorylation and ubiquitination of IFN receptor subunit 1 (IFNAR1) which then undergoes proteasome- and lysosome-dependent degradation [29]. Reduction of IFNAR1 on cell surface attenuates IFN signaling and innate antiviral responses.

## 3. Ubiquitin Regulates Influenza Virus Entry and Replication

The entry of viruses into animal cells follows a sequence of events including attachment of the virus particle to the cell surface, signaling, internalization, endocytic traffic, fusion, capsid uncoating, and genome release [30,31]. IAV binds to sialic acids on cell surface glycoproteins and glycolipids [32], which leads to lipid-raft clustering and activation of epidermal growth factor receptor (EGFR) and receptor tyrosine kinases that facilitate clathrin-mediated endocytosis and macropinocytosis [33,34,35].

The viral M2 channel opens in mildly acidic endosomes, allowing protons and potassium ions to enter the viral core [36,37]. This primes the core for uncoating by weakening core protein interactions. The importance of priming is signified by the low infection rate in acid-mediated bypass experiments (in which the virus enters the cytoplasm by direct fusion at the plasma membrane) compared to infection by the endocytic route [37]. In late endosomes (LEs) or mature macropinosomes, viral envelope glycoprotein HA undergoes an irreversible, low pH (5.5–5.0)-induced conformational change that activates its membrane fusion activity [38]. The viral envelope fuses with the limiting membrane of the endosome to form a fusion pore, through which the particle gains access to the cytosolic milieu for the first time during entry.

Ubiquitination plays a key role in IAV entry, especially during the step of uncoating. The itchy E3 ubiquitin protein ligase (ITCH) has been shown to promote IAV escape from LEs via the ubiquitination of M1 [39]. Uncoating is inhibited by the depletion of cullin 3 (Cul3), an E3 ubiquitin ligase that promotes endosome maturation [40,41]; the defects in the LE compartments in Cul3-depleted cells were reflected by a highly vacuolated LE/lysosome (LY) phenotype with large spherical, Rab7-positive, fluid-filled endosomes [40]. The endosomal sorting complexes required for transport (ESCRTs) mediate the sorting of ubiquitinated membrane proteins into intraluminal vesicles (ILVs) [42,43]. Depletion of components of the ESCRT machinery results in fewer ILVs and accumulation of cargo in endosomes with abnormal morphology [44,45,46].

Cul3 and the broad complex (BrC), tramtrack (Ttk) and bric-à-brac (Bab) (BTB)-adaptor speckle type BTB/pox virus and zinc finger (POZ) protein like (SPOPL) is responsible for the ubiquitination and degradation of the endosomal adaptor protein epidermal growth factor receptor pathway substrate 15 (EPS15) [47]. In the absence of SPOPL, EPS15 degradation and subsequent ILV formation are compromised, inhibiting EGFR degradation and IAV uncoating [40,47]. After uncoating via histone deacetylase 6 (HDAC6) and aggresome processing (discussed in Section 6), viral ribonucleoproteins (vRNPs) are released into the cytosol and trafficked to the nuclear pore complex (NPC) independent of microtubules or actin filaments [48,49,50]. They enter the nucleus via the activity of importin α/β where, in a complex with various co-opted host factors, the viral polymerase directs transcription and replication of the genome [51,52,53].

Post nuclear entry, the monoubiquitination of NP at residue K184 is crucial for virus RNA replication, possibly by regulation of NP–RNA interaction. Ubiquitination at K184 is counteracted by a host deubiquitinase USP11 [54]. Ubiquitination of viral polymerase and UPS activity promotes the activity of the polymerase, and ubiquitin expression leads to accumulation of vRNA, complementary RNA (cRNA) and viral mRNA [55]. Nonstructural protein 2 (NS2) of IAV binds to aminoacyl-tRNA synthase complex-interacting multifunctional protein 2 (AIMP2) which protects AIMP2 from K48-linked polyubiquitination and degradation. AIMP2 promotes nuclear export of vRNPs and IAV replication at late steps of infection. It also counteracts ubiquitination of M1 at K242 and subsequent proteasomal degradation by an unknown mechanism [56]. Modification of the same M1 K242 by a small ubiquitin-like modifier (SUMO) is crucial for nuclear export of newly produced vRNPs and viral morphogenesis [57], which could explain AIMP2-mediated enhancement of IAV replication at late steps of infection [56].

## 4. HDAC6 Binds to Unanchored Ubiquitin

**Figure 2:**
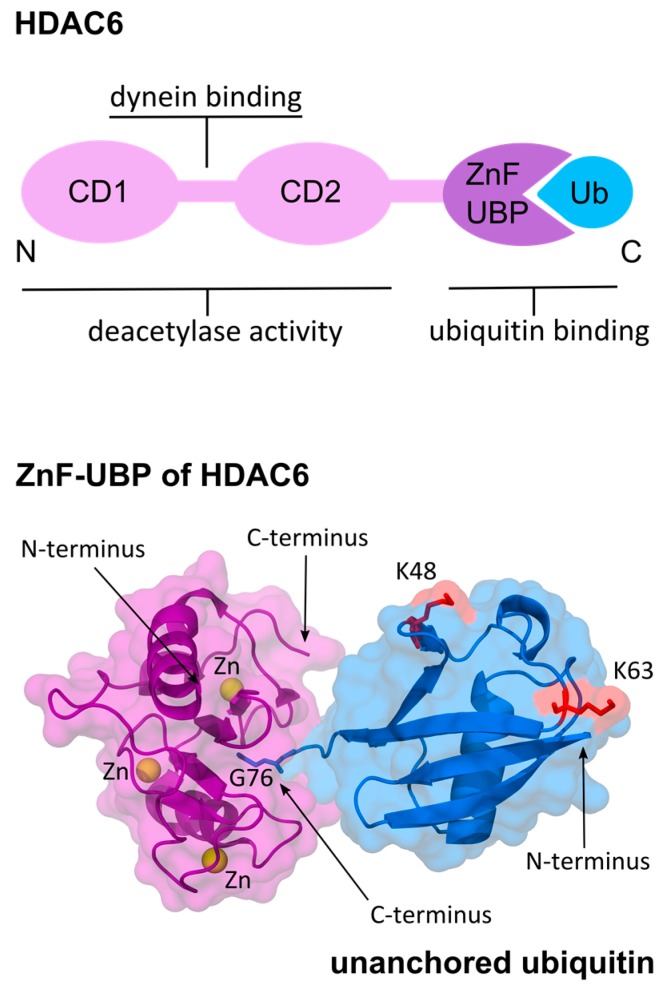
Functional domains of histone deacetylase 6 (HDAC6) and zinc finger ubiquitin-binding domain (ZnF-UBP) binding to ubiquitin (Ub) C-terminus. **Top**: Functional domain organization of HDAC6. HDAC6 contains tandem catalytic domains CD1 and CD2, as well as, a ZnF-UBP [63,65,72]. The linker sequence between the two deacetylase domains is responsible for interaction with dynein via p150^glued^ [66,67]; **Bottom**: Structure of the ZnF-UBP of human HDAC6 bound to ubiquitin (PDB ID: ubq1 [73] and 3gv4 [71]). The ZnF-UBP forms a deep pocket that specifically binds the C-terminal diglycine motif (G75, G76) of unanchored ubiquitin [70]. The coordination of zinc ions (Zn) in the ZnF-UBP, and K48, K63 residues of ubiquitin are shown.

HDAC6 is a cytoplasmic enzyme that promotes autophagic clearance of protein aggregates and protects cells from cytotoxic accumulation of misfolded aggregated proteins [58,59,60,61,62,63,64]. The two catalytic domains CD1 and CD2 of HDAC6 form together an ellipsoid-shaped complex of pseudo-two-fold symmetry [65]. Between the two catalytic domains there is a polypeptide that binds to the dynein motor [66,67]. Besides enzymatic activity, HDAC6 has an intrinsic ubiquitin-binding capability carried out by a zinc finger ubiquitin-binding domain (ZnF-UBP) close to the C-terminus (Figure 2), making it unique among HDAC family proteins [68].

Ubiquitin moieties in polyubiquitinated substrates are typically recognized by UBPs through their globular surface and hydrophobic core [69]. HDAC6 ZnF-UBP, on the other hand, has a unique deep binding pocket where ubiquitin’s C-terminal diglycine motif enters and binds with high affinity and specificity [70]. Thus, HDAC6 ZnF-UBP only binds to free mono ubiquitin and unanchored ubiquitin chains (Figure 2). Biochemical and structural analysis of the ZnF-UBP in complex with the full-length ubiquitin or the ubiquitin C-terminal RLRGG-peptide confirmed the necessity of the terminal diglycine for ZnF-UBP binding [63,71]. When ubiquitin is anchored to a target polypeptide via the C-terminus, HDAC6 ZnF-UBP cannot bind to it.

## 5. HDAC6 Regulates Aggresome Processing

**Figure 3:**
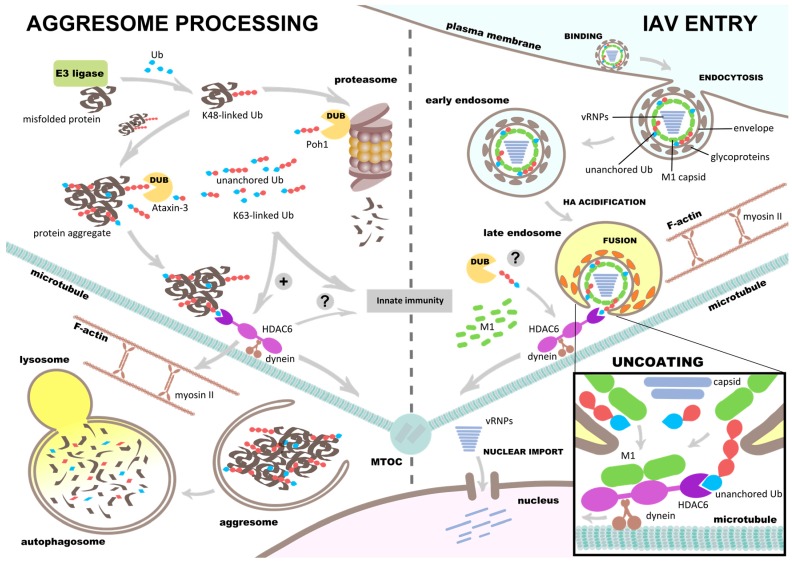
HDAC6 and unanchored ubiquitin promote aggresome processing and influenza A virus (IAV) uncoating. **Left side**: HDAC6 regulates the aggresome processing machinery. E3 ubiquitin ligases polyubiquitinate misfolded proteins, and those with a tetra K48-linked ubiquitin chain or longer are degraded by the proteasome [78]. Proteasome inhibition leads to formation of large, misfolded protein aggregates with polyubiquitinated chains, which are cleaved into unanchored ubiquitin chains by DUBs such as Ataxin-3. The C-terminus of ubiquitin is exposed on the unanchored ubiquitin chains and bind to HDAC6 ZnF-UBP. This, by yet unknown mechanisms, activates dynein binding of HDAC6 via its linker region between CD1 and CD2. The unanchored ubiquitin chain(s) is now linked to the dynein motor and microtubules, allowing retrograde transport of the protein aggregate toward the microtubule-organizing center (MTOC) (**aggresome formation**). Misfolded aggregates that are sequestered to the MTOC eventually form a large complex called the aggresome. HDAC6 promotes autophagy, which involves the actin network, myosin II, and Poh1, a proteasome-associated DUB. Poh1 generates K63-linked unanchored ubiquitin chains that activate HDAC6 (**aggresome clearance**) [12]. The **red** ubiquitin (Ub) depicts ubiquitin that is anchored, the **blue** ubiquitin depicts unanchored ubiquitin with a free C-terminus; **Right side**: IAV hijacks the aggresome processing machinery during host cell entry. After binding to the cell surface, IAV internalizes by endocytosis, travels to late endosomes (LEs) in the vicinity of the MTOC. In LEs the low pH (5.5–5.0) triggers hemagglutinin (HA) acidification and fusion of the viral envelope with the limiting endosomal membrane. The fusion pore exposes the viral core containing unanchored ubiquitin chains to the cytosol, which recruit HDAC6 and activate the aggresome processing machinery. HDAC6 binds to matrix protein M1, dynein, and myosin, and promotes capsid disassembly by the shearing force of the cytoskeletal motors (**box; uncoating**). M1 becomes dispersed in the cytosol, the viral ribonucleoproteins (vRNPs) penetrate into the cytosol and are imported into the nucleus through nuclear pore complexes by importin α/β [52,66]. Unanchored ubiquitin carried by IAV might activate HDAC6 similar to aggresome processing [12]. HDAC6, unanchored ubiquitin chains are implicated in retinoic acid-inducible gene 1 (RIG-I) immune signaling and NACHT, LRR and PYD domains-containing protein 3 (NLRP3) inflammasome regulation [13,79,80].

Misfolded proteins are tagged by polyubiquitin and targeted for degradation by proteasome activity. However, if clearance of misfolded proteins is insufficient, they can shape sizable aggregates that interfere with cell homeostasis [74]. How does HDAC6 ZnF-UBP recruit protein aggregates? Ouyang et al. [63] showed that unanchored ubiquitin chains with various lengths are found in misfolded protein bundles. Unanchored ubiquitin chains are generated in situ by Ataxin-3, an aggregate-associated, polyubiquitin-editing DUB that cuts within ubiquitin chains. Ataxin-3 exposes C-terminal diglycine motifs that serve as recognition tags for protein waste. These tags recruit HDAC6 and facilitate subsequent dynein-mediated transport of the aggregate to the microtubule-organizing center (MTOC) (Figure 3). How do the ubiquitin chains, despite being unanchored, remain associated with the aggregates? One possibility is via interaction of the ubiquitin hydrophobic patch with exposed hydrophobic cores of the misfolded proteins. In this scenario, ubiquitin chains would become trapped inside a net of aggregated polypeptides and cannot diffuse away.

Ataxin-3 preferentially cleaves K63-polyubiquitinated substrates [75], and when Ataxin-3 is depleted, HDAC6 is incapable of associating with protein aggregates [63]. Proteasomes do not degrade aggresomes but tend to congregate in their vicinity [12,76]. A proteasome-associated K63-specific DUB called Poh1 is necessary for aggresome clearance because it produces unanchored K63-linked ubiquitin chains which activate HDAC6 [12]. Heat shock protein 90 (Hsp90) is a binding partner of HDAC6 [77] and contributes to aggresome clearance by stimulating Poh1 to produce unanchored ubiquitin chains [76]. Ubiquitin binding to HDAC6 regulates the repressive Hsp90–heat shock factor 1 (HSF1) complex, the dissociation of which leads to activation of HSF1 and expression of cellular chaperones [58]. In Poh1-deficient cells, aggresome clearance is inhibited but can be restored by microinjecting unanchored K63-linked ubiquitin [12]. Toxic protein aggregates are processed by autophagy after being sequestered to the MTOC, and HDAC6 regulates the formation of a cortactin-dependent F-actin network that is interspersed among such protein aggregates. This activity stimulates autophagosome-LY fusion and aggresome degradation [61] (Figure 3).

## 6. Influenza Virus Uses Aggresome Processing for Capsid Uncoating

IAV X31 virions encapsidate a variety of unanchored ubiquitin moieties which include mono-, di-, tri-, tetra-, penta-, hexa-, and hepta-ubiquitin chains [66]. Following viral fusion at LEs, these ubiquitin moieties are exposed to the cytoplasm and attract HDAC6 to the sites of fusion on the surface of LEs. HDAC6 in turn binds to the capsid, which during endocytic transit has been broken down into M1 dimers [81], and links it to cytoskeleton motors dynein and myosin. This generates a shearing force that disassembles the capsid and releases the vRNPs into the cytoplasm (Figure 3). Point mutations H1094/H1098 or W1116 of the mouse HDAC6 ZnF-UBP inactivate ubiquitin binding to HDAC6 and block uncoating [66,82]. By blocking HDAC6 binding to dynein, dynein and myosin activity, or by depolymerizing microtubules or actin, uncoating is diminished [66]. It is likely that DUBs assist in the process of IAV uncoating. This is because DUBs such as ataxin-3 [63] and Poh1 [12] that generate unanchored ubiquitin chains are required for HDAC6-mediated aggresome processing.

**Figure 4:**
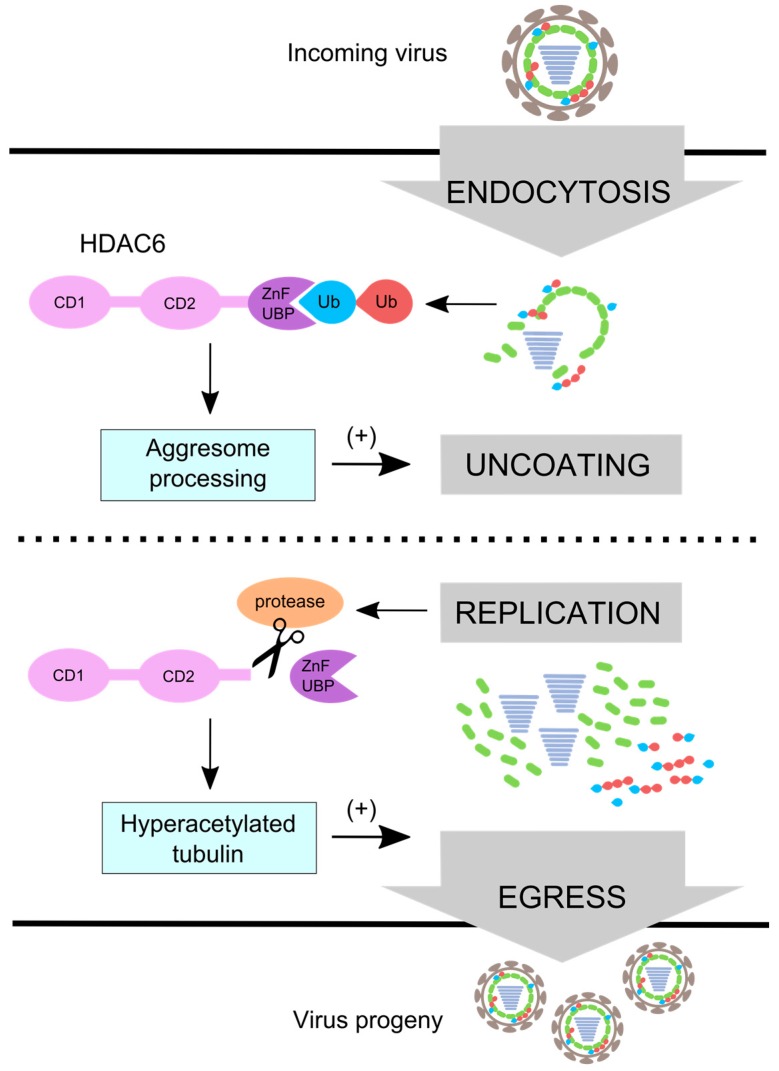
IAV uses HDAC6 differentially during entry and egress. IAV uses aggresome processing and unanchored ubiquitin chains in an HDAC6 ZnF-UBP-dependent manner to promote capsid uncoating during entry. During replication IAV induces ZnF-UBP cleavage by caspase 3 [83] which inactivates virus uncoating activity. Microtubules become hyperacetylated [59] and promote viral egress [90]. The **red** ubiquitin (Ub) depicts ubiquitin that is anchored, and the **blue** ubiquitin depicts unanchored ubiquitin with a free C-terminus.

What promotes virus uncoating during cell entry can potentially interfere with the assembly of newly replicated virions. To ensure proper assembly, viruses remove uncoating factors during the late stage of infection [30]. The IAV strategy is dually cunning; it not only inactivates HDAC6 but also induces a stable microtubule network that promotes viral progeny egress and budding. During replication, IAV cleaves off the HDAC6 ZnF-UBP by activating caspase 3 [83], which prevents premature capsid uncoating. HDAC6 inactivation combined with virus replication increases acetylation of microtubules. Acetylation promotes the association of microtubules with kinesin-1 and dynein motors which regulate endosome trafficking [84,85,86,87]. Cells lacking HDAC6 deliver EGFR prematurely to LE/LY compartments, resulting in faster EGFR degradation [88]. Hyperacetylated microtubules promote IAV genome egress via Rab11-positive recycling endosomes [89,90,91,92] (Figure 4). Although hyperacetylated microtubules appear to play no role in IAV uncoating [66], we cannot exclude the possibility that kinesin, in addition to dynein and myosin, contributes to the uncoating process.

Some viruses including poxvirus, African swine fever virus (ASFV), herpesviruses and retroviruses induce the formation of inclusion bodies near the MTOC. These so-called virus factories or viroplasms resemble aggresomes, and their contribution to virus replication and innate immune response has been discussed in detail by Wileman [93]. Interestingly, HDAC6 activates IFN-β [94] and regulates the RIG-I-mediated antiviral innate immune response. Deacetylation of RIG-I K909 by HDAC6 promotes viral RNA-sensing activity [80], and RNA viruses replicate better in immune cells depleted of HDAC6 due to reduction in IFN-β and proinflammatory cytokines [80]. However, the in vivo effects of IAV challenge in these mice remain to be determined.

## 7. Unanchored Ubiquitin Regulates RIG-I-Like Receptor Signaling

The role of innate immunity in RNA virus infection has been reviewed on many occasions [95,96,97], and we will only discuss some of the general aspects linked to ubiquitination. Pathogen-associated molecular patterns (PAMPs) of invading viruses are recognized by pattern recognition receptors (PRR). This leads to the activation of different signaling cascades, with the final production of IFNs the mediators of antiviral responses. Regulation of innate immune signaling relies on post-translational modifications including ubiquitination, and many viruses have evolved mechanisms to alter these ubiquitination events [14].

RIG-I, a member of the RIG-I-like receptor (RLR) family, is the most important PRR for viral detection and type I IFN production in infected epithelial cells. The two best-studied RLR members—RIG-I and melanoma differentiation-associated protein 5 (MDA5)—are cytoplasmic sensors critical for the detection of viral 5′-triphosphate single-stranded RNA (ssRNA) and long double-stranded RNA (dsRNA), respectively, which are generated after viral replication. Upon recognition of viral RNA, the helicase domain of RIG-I undergoes conformational changes that enable its caspase-recruitment domains (CARDs) to bind to mitochondrial antiviral signaling protein (MAVS). This finally leads to activation of nuclear factor kappa-light-chain-enhancer of activated B cells (NF-κB) and interferon response factor (IRF), and to the expression of type I IFNs [95,96,97]. K63-linked polyubiquitination of CARD by the E3 ubiquitin ligase TRIM25 induces conformational change and CARD tetramerization, which is necessary for signal transduction to MAVS [25].

Another E3 ubiquitin ligase, Riplet, was shown to regulate RIG-I through K63-linked ubiquitination [98,99]. Recent reports suggest that not only covalently attached but also unanchored K63-linked polyubiquitin chains activate RIG-I signaling. However, according to structural studies, unanchored ubiquitin chains stabilize the signaling-active RIG-I tetramer less efficiently than covalent K63-ubiquitin [100]. IRF3 is activated by in vitro-generated K63-polyubiquitin chains that bind to RIG-I [13], but the role of unanchored K63-linked ubiquitin in MDA5-mediated IRF3 activation is not clear cut [101,102].

## 8. Regulation of RIG-I by Ubiquitin Is Species-Specific

Influenza pandemics can occur when zoonotic influenza viruses adapt for efficient replication and transmission in humans. The incompatibility of avian IAV strains with the human host can be explained by the lack of factors supporting viral replication in human cells or the presence of restriction factors that cannot be counteracted by avian strains until adaptive mutations are selected [103]. It has been known for some time that in human cells, avian-derived IAV polymerase is poorly active. In heterokaryons formed between human and avian cells, the activity of avian IAV polymerase is restored [104], and the responsible host factor co-opted in avian cells was recently identified as ANP32A [105].

While in humans activation of RIG-I by unanchored ubiquitin seems to be only an accessory mechanism to covalent ubiquitination, in ducks, the natural host of IAV, unanchored ubiquitin may play the main role in RIG-I signaling. It has been shown that duck TRIM25 can activate RIG-I CARD mutant lacking lysine residues by a mechanism independent of ubiquitination [106]. TRIM25 produces unanchored ubiquitin chains that activate RIG-I in vitro [13], suggesting that interaction with unanchored ubiquitin might regulate duck RIG-I. Adaptation of IAV strains to counteract different immune responses in avian and human hosts likely contributes to interspecies transmission and pandemic risk.

## 9. Ubiquitin Regulates Inflammasomes

RIG-I activates inflammasomes, the key components of the innate immune response to pathogens or tissue damage [107]. These large multiprotein complexes are formed rapidly in response to PAMPs and serve as scaffolds to activate caspase 1, which processes pro-interleukin (IL)-1β and pro-IL-18 into IL-1β and IL-18, respectively, which can then be secreted [95,107]. Nucleotide-binding oligomerization domain (NOD)-like receptor family member NACHT, LRR and PYD domains-containing protein 3 (NLRP3) inflammasome is involved in defense against infection with IAV and many other viruses including hepatitis C virus [107,108,109,110,111]. Ubiquitination and deubiquitination regulates NLRP3 inflammasome activation. For example, breast cancer 1 (BRCA1)/breast cancer 2 (BRCA2)-containing complex subunit 3 (BRCC3) deubiquitinates NLRP3, and pharmaceutical inhibition of DUBs restricts inflammasome activation [79,112,113], whereas E3 ubiquitin ligases LUBAC and TRIM33 promote inflammasome assembly [114,115]. In bone-marrow-derived macrophages (BMDMs) depleted of HDAC6, there is a significant boost in caspase 1 activation and IL-1β secretion as a response to NLRP3 activation. HDAC6 ZnF-UBP interaction with NLRP3 inhibits inflammasome activation [79], suggesting that unanchored ubiquitin regulates this pathway. NS1, the major suppressor of the IAV IFN response, antagonizes both TRIM25/Riplet-mediated RIG-I ubiquitination and NLRP3 inflammasome activation which underscores their significance in anti-viral immunity [28,108,116].

## 10. Future Perspectives

Incorporation of host cellular proteins into or onto the newly formed enveloped viruses is a known phenomenon. A few of such virus-incorporated host proteins have been described, especially in retroviruses [117]. IAV virions produced in different hosts contain a wide range of host-derived proteins as detected by mass spectrometry [118,119]. Among these are ubiquitin, cytoskeletal proteins, and tetraspanin cluster of differentiation (CD) 81 [118,119,120]. The biological significance of these non-viral proteins is largely unknown; encapsidated enzymes, for example, could play a regulatory role in IAV entry. In order to better understand how HDAC6 contributes to IAV uncoating, the following aspects should be addressed: (1) virion ubiquitin content (e.g., abundance of unanchored moieties and their linkages); (2) architecture of the viral fusion pore; and (3) availability of other uncoating factors in the cytoplasm.

Can viral ubiquitin be used as a predictor of IAV infectivity? Does unanchored ubiquitin regulate the immune response during IAV entry? It is intriguing that K63-linked ubiquitin chains are implicated in aggresome clearance, as this modification is an element of immune signaling [121] rather than the UPS (often K48-linked). There is perhaps an unexplored link between ubiquitin, aggresome processing, and immune response to viral infection. Unraveling such molecular interactions between virus and host cell is of critical importance for understanding the cell biology of virus infection, and for future design of antivirals.
